# Low-Frequency Electrical Stimulation Promotes Satellite Cell Activities to Facilitate Muscle Regeneration at an Early Phase in a Rat Model of Muscle Strain

**DOI:** 10.1155/2021/4218086

**Published:** 2021-02-06

**Authors:** Da-an Wang, Qing-zheng Li, Dong-ming Jia

**Affiliations:** ^1^School of Physical Education and Health, Hainan Tropical Ocean University, Sanya 572022, China; ^2^China Institute of Sport Science, Beijing 100763, China; ^3^Zhejiang Police Vocational College, Hangzhou 310018, China

## Abstract

The capability of regeneration for skeletal muscle after injury depends on the differentiation and proliferation ability of the resident stem cells called satellite cells. It has been reported that electrical stimulation was widely used in clinical conditions to facilitate muscle regeneration after injury, but the characterization of satellite cell responses to the context of low-frequency electrical stimulation in early-phase muscle strain conditions has not been fully clarified. In this study, we aim to investigate the effects of low-frequency electrical stimulation (frequency: 20 Hz; duration: 30 minutes, twice daily) on satellite cell activities in a rat model for the early phase of muscle strain. Firstly, we adopted our previously developed rat model to mimic the early phase of muscle strain in human. After then, we examined the effects of low-frequency electrical stimulation on histopathological changes of the muscle fiber by hematoxylin and eosin (H&E) staining. Finally, we investigated the effects of low-frequency electrical stimulation on satellite cell proliferation and differentiation by quantification of the expression level of the specific proteins using western blot analyses. The muscle strain in biceps femoris muscles of rats can be induced by high-speed rotation from knee flexion 50° to full knee extension at 960°·s^−1^ angular velocity during its tetany by activating the sciatic nerve, as evidenced by a widening of the interstitial space between fibers, and more edema or necrosis fibers were detected in the model rats without treatment than in control rats. After treatment with low-frequency electrical stimulation (frequency: 20 Hz; duration: 30 minutes, twice daily), the acute strained biceps femoris muscles of rats showed obvious improvement of histomorphology as indicated by more mature muscle fibers with well-ordered formation with clear boundaries. Consistently, the expression levels of the MyoD and myogenin were marked higher than those in the rats in the animal model group, indicating increased satellite cell proliferating and differentiating activities by low-frequency electrical stimulation. This study shows that low-frequency electrical stimulation provides an effective stimulus to upregulate the protein expression of MyoD/myogenin and accelerate the restoration of structure during the early phase of muscle strain. This may have significance for clinical practice. Optimization of low-frequency electrical stimulation parameters may enhance the therapeutic outcome in patients.

## 1. Introduction

Muscle injuries are the most common injuries sustained by athletes. The high prevalence of muscle strains is well documented in sports, for example, football [1], soccer [2], cricket [3], and rugby [4]. Muscle strains are a result of muscle fiber tears due to overstretching, which are not only restricted to professional athletes but also happen to amateur and recreational athletes. Even a mild muscle strain can cause a delay in returning to sport or even lead to a failure in the sporting activity one is able to do. Rehabilitation of injured skeletal muscle is an area that continues to present a challenge for the athletes and sports medicine clinicians.

Skeletal muscles exhibit a high plasticity for morphological and functional adaptations to growth, training, mechanical overload, and injury. The processes by which these adaptations occur are largely dependent on a small population of satellite cells, which are resident between the basal lamina and plasma membrane of the muscle fibers. In response to muscle injury, these cells are activated to self-renew, proliferate, then differentiate into myoblasts, and finally fuse with one another to support muscle regeneration [5–7]. Electrical muscle stimulation has received increasing attention in the last few years, because it has the potential to regulate the satellite cell activity [8, 9]. Electrical muscle stimulation is the elicitation of muscle contraction using electric impulses, which mimic the action potential from the central nervous system. In clinical conditions, electrical stimulation can be used to improve muscle action and performance such as increasing muscle strength [10], improving range of motion [11], decreasing atrophy [8], and decreasing pain [12]. However, the characterization of muscle satellite cells and healing of strained muscle responses in the context of low-frequency electrical stimulation in early-phase muscle strain conditions have not been fully clarified. Accordingly, the aim of this study was to investigate the effects of low-frequency electrical stimulation (frequency: 20 Hz; duration: 30 minutes, twice daily) on satellite cell activity and restoration of structure during the early phase of biceps femoris muscle strain.

## 2. Materials and Methods

### 2.1. Animals

Thirty-six male Sprague-Dawley (SD) rats (300-400 g body weight (bw)) were utilized in this study. All the rats were housed in the Animal Center of the Hong Kong Polytechnic University in a thermally controlled room maintained at 22°C with 12 : 12 h dark-light cycles. The experiments were conducted in accordance with the National Institutes of Health Guide for the Care and Use of Laboratory Animals and were approved by the Hong Kong Polytechnic University committee on Care and Use of Laboratory Animals.

### 2.2. Experimental Design

All the rats were assigned randomly into one of six groups as follows: control group (*n* = 6) and animal model groups—D0 group (*n* = 6), D7 group (*n* = 6), D14 group (*n* = 6), D7-20 Hz group (*n* = 6), and D14-20 Hz group (*n* = 6). The rats in D0, D7, and D14 groups were sacrificed at days 0, 7, and 14 after strain induced without any treatment. The rats in D7-20 Hz and D14-20 Hz groups will be subjected to low-frequency electrical stimulation (frequency: 20 Hz; duration: 30 minutes, twice daily) for 3 and 10 days, respectively. After sacrifice, the biceps femoris muscles were dissected for histological and western blot analysis.

### 2.3. Establishment for the Animal Model of Muscle Strain

A novel system was designed to create a partial biceps femoris strain [13]. This system consists of an AC servomotor system, high-performance Stepper/Servo Motion Controllers, a torque sensor, an electrical stimulator, and an amplifier. One end of the torque sensor was linked to the motor arm. The torque sensor, with an adjustable plate attached to the other torque sensor arm, measures the knee torque during muscle activation. All rats except those in the control group were anesthetized with a subcutaneous injection of ketamine-xylazine cocktail (100 and 5 mg/kg body mass, respectively). One limb was chosen by random to create a partial biceps femoris strain. The animals were placed in the test apparatus, and a strap was wrapped around the waist to ensure no slippage with the pelvis in the neutral position. Cares were taken to ensure that the animal was positioned so that the axis of knee joint rotation was aligned with the axis of rotation of the motor. The limb was moved from knee flexion 50° to full knee extension at 960°·s^−1^ angular velocity and then returned to the starting position. When the knee was rotated into knee extension, the biceps femoris was stimulated to tetany by activating the sciatic nerve via subcutaneous needle electrodes, which were placed over the sciatic nerve in the region of the ischial tuberosity. Twitch threshold voltages below ~1 V indicated accurate electrode placement. If threshold voltages were too high, electrodes should be repositioned to achieve optimal stimulation. Then, stimulation voltages would increase until the peak twitch torque was reached. Subsequent torque measurements should be made at two to three times this level to ensure complete activation of the biceps femoris.

### 2.4. Electrical Stimulation Protocol

To achieve electrical stimulation, the rats in the D7-20 Hz group and D14-20 Hz group were anesthetized with a subcutaneous injection of ketamine-xylazine cocktail (100 and 5 mg/kg body mass, respectively) at the fifth day. The hindlimb to be stimulated was shaved, and two electrodes (1 cm × 2 cm) were placed on the skin overlying the middle of the hamstring and the lateral side of the fibula. Both electrodes were secured in place with ventilated tape. Insulating materials were placed between the two electrodes. Stimuli (200 *μ*s pulse duration, 5 s on and 10 s off, 20 Hz) were applied twice a day for 30 minutes with a 4-hour rest between treatments. There was a total of 1 hour of treatment per day using a DC-driven electrical stimulator (CEFAR Digital Unit, Lund, Sweden). The intensity of stimulation was set to induce visible muscle contractions. The treatment was given to the D7-20 Hz group and D14-20 Hz group for 3 and 10 days, respectively. No interventions were given to the D0 group, D7 group, and D14 group.

### 2.5. Histological Analysis

Histopathological evaluations were conducted on rats of the control group, D0 group, D7group, D7-20 Hz group, D14 group, and D14-20 Hz group at intervals (0, 0, 7, 7, 14, and 14 days postinjury, respectively). The rats were anesthetized with a subcutaneous injection of ketamine-xylazine cocktail (100 and 5 mg/kg body mass, respectively) and killed by cervical dislocation. H&E staining was performed on sections to examine the general morphology of the fiber. The biceps femoris muscles were dissected and frozen in isopentane cooled by liquid nitrogen, embedded in OCT (Miles Scientific, Elkhart, IN, USA), and stored at -80°C for histological and immunohistochemical processing. Using a microtome cryostat at –20°C, 10 cross sections (10 *μ*m thick) were cut at each of six levels equally spaced along the length of the biceps femoris. Sections at each level were stained using routine H&E staining.

### 2.6. Western Blot Analysis

Frozen muscles were homogenized in a solution containing 4% sodium dodecyl sulfate (SDS), 50 mM Tris-HCl (pH 6.8), 1 mM phenylmethylsulfonyl fluoride (PMSF), and 1 *μ*M each of leupeptin and pepstatin A. The homogenates were centrifuged at 4°C for 10 min at 14k rpm, and the supernatants were collected into new tubes. The protein concentration in each sample was measured with a spectrophotometer by using the detergent-compatible protein assay (Bio-Rad, Hercules, CA, USA). Equal amounts of protein from each sample was mixed with loading buffer and run on 10% sodium dodecyl sulfate-polyacrylamide (SDS-PAGE) gels with low voltage (60 V) using gel electrophoresis. The protein molecules were separated according to their sizes and transferred onto the blotting membranes. After transfer, the blotting membranes were blocked with 5% skim milk in TBS for one hour and then added primary antibody mouse monoclonal anti-myoD (5.8A, 1 : 200 dilution; Santa Cruz Biotechnology) or mouse monoclonal anti-myogenin (F12B, 1 : 200 dilution; Sigma) in 5% bovine serum albumin (BSA) and incubated overnight at 4°C on a shaker. Following incubation, the membranes were washed with TBST at room temperature and incubated again with a secondary antibody in 5% skim milk in TBST for two hours. A cassette was used to visualize the result in the dark room. The bands were scanned into a computer, and images were stored in a TIFF format. The intensity of the bands of MyoD and myogenin proteins was quantified by using the NIH Image software. The protein levels of MyoD and myogenin in the biceps femoris were expressed relative to the protein level of *α*-tubulin.

### 2.7. Statistical Analysis

Between-group comparisons were performed using a one-way analysis of variance (ANOVA) with the Bonferroni post hoc analysis. If data exhibited a nonnormal distribution (assessed by the Shapiro-Wilk test), nonparametric Kruskal-Wallis statistics was used to detect the differences between the groups. All data were presented as means ± standard error of the mean (SEM). Statistical significance was set at *P* < 0.05.

## 3. Results

### 3.1. Histomorphological Changes of Muscle Strain in the Animal Model

**Figure 1:**
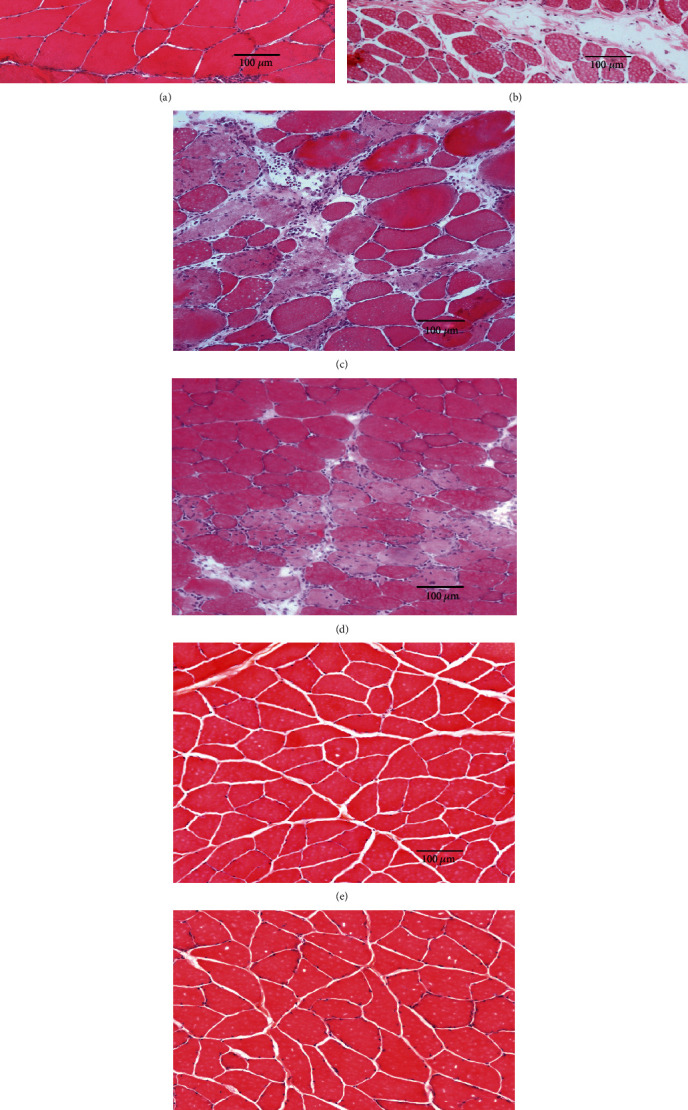
Hematoxylin and eosin staining of cross sections of the biceps femoris muscle. Scale bar = 100 *μ*m. (a) Control group, (b) D0 group, (c) D7 group, (d) D7-20 Hz group, (e) D14 group, and (f) D14-20 Hz group.

H&E staining indicated that normal fibers with polygonal aspects are multinucleated syncytia with their nuclei located at the periphery as seen in the biceps femoris muscle cross section (Figure 1(a)). After injury, H&E staining indicated fibers' structural disorder and hemorrhage in the acute strained biceps femoris. Fibers in the acute strained biceps femoris had lost their normal polygonal shape. As demonstrated by a widening of the interstitial space between fibers, edema and necrosis were evident in the acute strained biceps femoris. There was a modest influx of inflammatory cells that was restricted to the damaged region (Figure 1(b)).

### 3.2. Increased Regenerating Muscle Fibers in the Biceps Femoris Muscle after Electrical Stimulation

**Figure 2:**
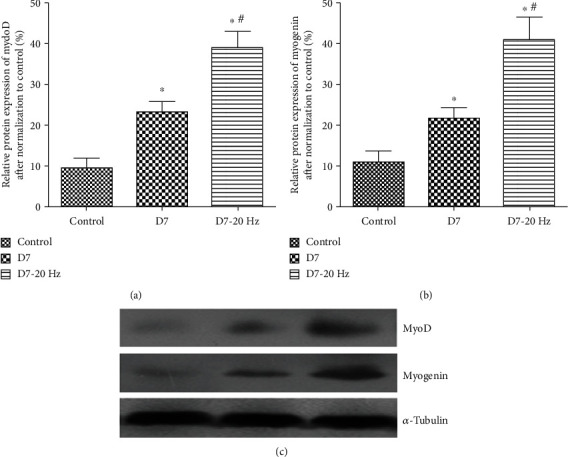
Western blot analysis of MyoD/myogenin protein expression at 14 days postinjury. Histogram (a, b: values are means ± SEM) and representative western blots (c) showing relative levels of MyoD/myogenin protein expression in biceps femoris muscles from control group, D14 group, and D14-20 Hz group rats (MyoD-specific band with molecular weight ~43–45 kDa, myogenin-specific band with molecular weight ~32–34 kDa, and *α*-tubulin-specific band with molecular weight 50 kDa). *α*-Tubulin was included as a loading control. ∗ indicates significant difference with the control (*P* < 0.05). # indicates significant difference with the D14 group (*P* < 0.05).

At 7 days after injury, new regenerating fibers with centrally located 5nuclei were detected. Disorganized muscle fibers of various sizes exhibited rounded contours, variable stain intensities, weakly stained nuclei, and greater interfiber distances (Figure 1(c)). Given low-intensity electrical stimulation in the D7-20 Hz group, many new regenerating muscle fibers were identified by centronucleation. Compared to the D7 group, the new fibers in the D7-20 Hz group became more mature and demonstrated well-ordered formation with clear boundaries (Figure 2(d)). Compared to the D14 group, the muscle fibers in the D14-20 Hz group had bigger cross-sectional areas at 14 days after injury (Figures 1(e) and 1(f)).

### 3.3. Elevated Protein Expression of MyoD and Myogenin in the Biceps Femoris Muscle after Electrical Stimulation

**Figure 3:**
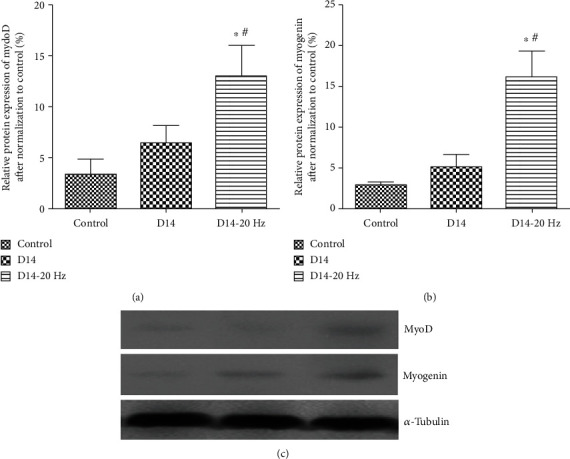
Western blot analysis of MyoD/myogenin protein expression at 7 days postinjury. Histogram (a, b: values are means ± SEM) and representative western blots (c) showing relative levels of MyoD/myogenin protein expression in biceps femoris muscles from control group, D7 group, and D7-20 Hz group rats (MyoD-specific band with molecular weight ~43–45 kDa, myogenin-specific band with molecular weight ~32–34 kDa, and *α*-tubulin-specific band with molecular weight 50 kDa). *α*-Tubulin was included as a loading control. ∗ indicates significant difference with the control (*P* < 0.05). # indicates significant difference with the D7 group (*P* < 0.05).

At 7 days postinjury, the levels of MyoD (23.1% ± 6.0%, Figure 3(a)) and myogenin (21.9% ± 6.1%, Figure 3(b)) protein expression increased in biceps femoris muscles of the D7 group, which demonstrated a significant difference at *P* < 0.05 compared to the control group (MyoD: 9.4% ± 5.2%, Figure 3(a); myogenin: 10.9% ± 6.1%, Figure 3(b)). When subjected to electrical stimulation, protein expression of MyoD (38.9% ± 8.8%, Figure 1(a)) and myogenin (41.2% ± 12.4%, Figure 1(b)) was greatly increased in the D7-20 Hz group compared to the control group and D7 group (*P* < 0.05). The levels of a MyoD-specific band with molecular weight ~43–45 kDa and a myogenin-specific band with molecular weight ~32–34 kDa were higher in the D7-20 Hz group at *P* < 0.05 compared to the control group and D7group (Figure 3(c)).

At 14 days postinjury, the levels of MyoD (6.5% ± 3.2%, Figure 2(a)) and myogenin (5.2% ± 3.4%, Figure 2(b)) protein expression decreased in the D14 group, although they were still higher than those in the control group (MyoD: 3.4% ± 3.2%, Figure 2(a); myogenin: 2.9% ± 0.6%, Figure 2(b)). No significant differences were observed between the control group and D14 group (*P* > 0.05). Electrical stimulation induced a marked increase in MyoD (13.1% ± 6.7%, Figure 2(a)) and myogenin (16.2% ± 7.4%, Figure 2(b)) protein expression in the D14-20 Hz group (*P* < 0.05) compared to the control group and D14 group. The levels of a MyoD-specific band with molecular weight ~43–45 kDa and a myogenin-specific band with molecular weight ~32–34 kDa were still higher in muscles of D14-20 Hz group rats at *P* < 0.05, compared with control group and D14 group rats (Figure 2(c)).

## 4. Discussion

In this study, we have explored the effectiveness of low-frequency electrical stimulation (frequency: 20 Hz; duration: 30 minutes, twice daily) on satellite cell activity and restoration of structure in the strained biceps femoris muscles during the early phase of muscle strain. The satellite cell activities and histopathological changes in the biceps femoris undergoing an acute muscle strain were determined using western blot analyses and histopathological evaluation, respectively. We have demonstrated that low-frequency stimulation greatly upregulated the protein expression of proliferating (MyoD) and differentiating (myogenin) satellite cells and accelerated the restoration of structure in new regenerated fibers during the early phase of biceps femoris muscle strain.

A previous study showed that the sprouting of new capillaries in mobilized muscles occurred more rapidly and intensively than that in injured muscles treated by immobilization during the early phase of muscle injury. The tissue repair was directly correlated with vascular ingrowth especially during the first week. What is more, as studied histologically, early mobilization induces better regeneration of muscle fibers and more parallel orientation of regenerating myofibers in comparison to immobilization [14]. However, it has been shown that (i) a larger connective tissue scar ensues and (ii) rerupture at the site of the original muscle trauma is common if active mobilization of the injured muscle is begun immediately after the injury. In fact, a certain period of immobilization (about five days for rat muscle) is required to allow newly formed granulation tissue to cover the injured area. However, if immobilization continued too long, it leads to contraction of the scar and to poor structural organization of the components of regenerating muscle and scar tissue [15]. Electrical muscle stimulation is the elicitation of muscle contraction using electric pulses. We hypothesized that gentle stretching caused by low-intensity electrical stimulation would make new regenerative muscle fibers grow along the stress lines, which were paralleled with the original muscle.

Therefore, we started low-intensity electrical stimulation at the fifth day after muscle strain. We used an electrical stimulator to generate electric impulses, which were delivered through surface electrodes on the skin paralleled with the original biceps femoris. The frequencies of electrical stimulation used vary widely depending upon the goals of intervention, but 20-50 Hz patterns are usually used for optimal results in most clinical conditions [16, 17]. In this study, we chose to apply low intensity of electrical stimulation at a frequency of 20 Hz because constant low-frequency stimulation produces a smooth contraction at low force levels [18], which may prevent reoccurrence of muscle injuries. Stimuli (200 *μ*s pulse duration, 5 s on and 10 s off, 20 Hz) were applied twice a day for 30 minutes with a 4-hour rest between treatments. The intensity of stimulation was set to induce visible muscle contractions, which made sure that the injured muscle was gently stretched to prevent the reruptures at the injury site. Electrical frequency is stated in the SI (International System of Units) unit of hertz (Hz), which refers to the electric pulses produced per second during stimulation (e.g., 1 Hz means that pulse repeats once per second).

As shown in this study, hematoxylin and eosin staining indicated that fibers in the acute strained biceps femoris lost their normal polygonal shape. As demonstrated by the structural disorder, widening of the interstitial space between fibers was evident in the acute strained biceps femoris. Given low-intensity electrical stimulation, new regenerating muscle fibers were identified by centronucleation and the new fibers became more mature and demonstrated well-ordered formation with clear boundaries. The orientation of the new regenerating fibers was more parallel with the original biceps femoris fibers in comparison to unstimulated muscles (Figure 1(d)).

In this study, we explored the effectiveness of low-frequency electrical stimulation on satellite cell activity using a panel of antibodies that recognize proteins uniquely expressed in satellite cells during the proliferating and differentiating stage. We made use of MyoD, a marker of myogenic proliferation [19–21], to identify the status of satellite cell proliferation. We also used antibodies specific for myogenin to examine active satellite cells upon electrical stimulation after muscle strain. Myogenin is regarded as one of the markers for satellite cell differentiation [6, 22, 23]. Our findings agree with previous studies that have shown the activation of satellite cells upon muscle injury resulting from direct injury to the muscle [19, 24]. As demonstrated in this study, low-intensity electrical stimulation after the muscle strain could accelerate the proliferation and differentiation of myogenic cells and the expression of their regulating factors MyoD and myogenin. This suggests that low-intensity electrical stimulation in the early phase (5 days postinjury) could accelerate recovery of strained muscles by enhancing satellite cell activity.

The present study has produced two important conclusions. First, it has shown that low-frequency electrical stimulation greatly accelerates the restoration of structure during the early phase of muscle strain. Second, low-frequency electrical stimulation provides an effective stimulus to upregulate the protein expression of MyoD/myogenin during the early phase of muscle strain. Since low-frequency stimulation greatly upregulates the protein expression of satellite cell activity and accelerates restoration of structure during the early phase of muscle strain, this may therefore have significance for clinical practice. Future researches are needed to optimize low-frequency electrical stimulation parameters to achieve the desired responses.

## Data Availability

The data used to support the findings of this study are available from the corresponding author upon request.
